# Environmental Justice: Where It Has Been, and Where It Might Be Going

**DOI:** 10.1146/annurev-publhealth-071621-064925

**Published:** 2023-01-09

**Authors:** Merlin Chowkwanyun

**Affiliations:** Department of Sociomedical Sciences, Mailman School of Public Health, Columbia University, New York, NY, USA

**Keywords:** environmental justice, environmental racism, racial health disparities, structural racism, health advocacy, health activism

## Abstract

Taking stock of environmental justice (EJ) is daunting. It is at once a scholarly field, an ongoing social movement, and an administrative imperative adopted by government agencies and incorporated into legislation. Moreover, within academia, it is multidisciplinary and multimethodological, comprising scholars who do not always speak to one another. Any review of EJ is thus necessarily restrictive.

This article explores several facets of EJ activism. One is its coalitional and “inside-outside” orientation. EJ activists are constantly forming alliances with other stakeholders, but these coalitions do not flout the importance of engaging with formal institutions. The review next turns to one set of such institutions—the courts and regulatory agencies—to see how well EJ claims have fared there. I then survey scientific findings that have been influenced by EJ. The review concludes with future directions for activists and scholars to consider: the changing nature of EJ coalitions, fragmentation within EJ and with other fields, the historical roots of environmental injustice, and opportunities for stronger infusion of the EJ lens.

## INTRODUCTION

Overviews of environmental justice (EJ), often written by major movement participants, commonly employ a foil: mainstream environmentalism of the 1970s. Writers note familiar lacunae: the latter’s focus on natural resource conservation, its white middle-class composition, its obliviousness toward the racialized impacts of environmental risks, and the secondary emphasis it placed on human health (versus environmental protection more broadly) (18–20). But then in the early 1980s came a more holistic environmentalism. Unlike environmentalist predecessors, EJ activists made the unjust differential distribution of environmental health risks—typically by race and social class—central to their work. They engaged in on-the-ground confrontation and publicized findings on disturbing spatial patterns identified by high-profile reports (119, 122).

Such a narrative then catalogs major wins in scattered localities but also beyond them, especially in the legal and administrative arenas, where EJ activists characterized unequal environmental burdens as civil rights violations. One landmark triumph is Executive Order 12898 (in 1994), signed by President Bill Clinton, which ordered federal agencies to collect data on the disparate “human health or environmental effects” of their programs and to take actions to mitigate them (121). The narrative concludes with a canvass of recent developments: more evidence of enduring patterns but also novel community-based research and the movement’s continued traction, including its influence on agency operations and policy making.

This review does not attempt to retell or extend that familiar narrative. Nor does it try to cover every crevice of a booming literature that itself has already been the topic of previous reviews and monograph-length handbooks and syntheses (17, 18, 60, 88, 114). Instead, I begin with the observation that environmental justice is both a term around which activists rally and one that characterizes scholarship. It crosscuts domains: not just activists and academics, but increasingly, government agencies and public policies. What results is a literature that has a daunting sprawl. The problem is compounded by the multimodal nature of EJ, which draws in not just those in public health but also multiple social sciences and humanities disciplines, to say nothing of other practitioner fields, ranging from law to urban planning, all of which do not always directly speak to one another.

Any review of EJ is thus necessarily restrictive. I begin by exploring EJ’s coalitional orientation, whereby EJ activists form alliances with other stakeholders. Yet however grassroots or “bottom up” they might be, these coalitions tend to also simultaneously engage formal institutions, not flout them. I focus in particular on courts and regulatory agencies to see how well EJ lawsuits and claims-making have fared there. I then turn to scientific findings influenced by EJ heuristics. The review concludes with four future directions for both activists and scholars.

I offer two final notes. I use the term activist capaciously—and perhaps unsatisfactorily—to describe anybody who promotes, via a variety of tactics, efforts to reduce inequalities in environmental health exposures, outcomes, and mitigation. In this broad conception, it means that someone who works in a nonprofit organization fighting for these causes is as much an activist as someone engaged in confrontational direct action at a protest before a chemical plant. In turn, the review takes note of different strategies adopted by the EJ movement and the gamut of settings it has entered. Second, to focus its inquiry, this literature review restricts itself to the United States. I address the limits of this focus—shared by many American EJ activists themselves—later in the review.

## THE EJ “INSIDE-OUTSIDE” STRATEGY AND COALITIONS

In early stages of activism, EJ activists often work outside formal institutions. They distribute ephemera, engage in door-to-door rallying of residents, organize community meetings, and participate in direct action confrontation. But unlike other movements, EJ activists have not eschewed formal institutions. A striking feature of EJ activism, in fact, is that it employs extra-institutional activism to accrue more formal recognition, credibility, and legitimacy. Much EJ activism thus utilizes an inside-outside strategy. Participants simultaneously agitate outside institutions even as they engage arms of the state (elected officials, regulatory agencies) and organizations like research universities. These processes have been documented most effectively in local case studies, where authors follow activists via participant observation and conduct extensive interviews and document analysis (6, 14, 25, 37, 61, 68, 94, 113, 115).

One inside-outside technique is coalition building. Coalitions reflect public foment, which in turn catalyzes constituent feedback loops and responsiveness from the state. Some writers have found that EJ itself can serve as a useful issue that unites otherwise disparate groups, particularly in multiracial coalitions (25, 70, 97). EJ activists have also united with those in such adjacent areas as transportation and spurred broad cross-sectoral policy efforts as a result. In the transportation case, it is unfair siting practices in the clustering of diesel bus depots or in the direction of traffic routes that has spurred broad cross-sectoral activism and policy as a result. (66, 67, 78, 113). EJ coalitions anticipated, by at least a couple decades, the current interest in health in all policies (HiAP), a framework that highlights the health implications of nonhealth stakeholders’ activities and that has been adopted by a number of local governments (51). It has presaged, too, a more recent parallel initiative, equity in all policies (EiAP), which centers equity concerns across disparate sectors (16).

Coalitions are not problem free. Case studies of EJ have delved into the fraying of coalitions—and local EJ campaigns more generally—to offer a list of problems for EJ activists to anticipate. These problems include tensions over the proper scope of EJ activism (e.g., single local battle versus more systemic reform), acceptable levels of policy compromise and political bargaining, rhetorical framing and messaging, the racial and class composition of EJ activists, and the reliance on limited grants (26, 57, 81, 94, 96, 97, 113). One example of these tensions comes from an influential account of battles over waste disposal in the Chicago metropolitan area during the 1980s and 1990s. There, some members of a coalition—and, crucially, elected officials with whom they had engaged—supported limited presence of waste incinerators because of perceived positive trade-offs: energy, jobs, and revenue. In another episode, a coalition uncritically supported the creation of a recycling plant in a predominantly low-income and minority neighborhood, introducing emissions and exposing a predominantly minority workforce to hazardous working conditions. The universal appeal of recycling to large swaths of the public—across racial and class lines—obscured the burdens that recycling facilities still posed to areas that disproportionately host them (94). This particular conundrum of coalitions extends to rhetoric. Another case study of EJ in Augusta, Georgia, notes that leaders switched between framing hazards as being universal versus of particular concern to African Americans, depending on context (26). The Augusta case maps onto general tension over whether EJ goals ought to be framed with that term versus the more pointed “environmental racism.”

Scholars have begun systematically examining the reasons for success and failure of coalitions. One analysis of 50 EJ coalitions showed that sympathetic lawmakers, especially at the state and federal level; rhetorical support from prominent national organizations (environmental and otherwise); and litigation all resulted in a higher chance of a successful outcome (57). This analysis affirmed single-site studies that have consistently found a high degree of effective engagement with government officials at all levels of government. It suggests two fruitful avenues for future inquiry: more macrolevel canvasses of accumulated EJ wins (alongside local case studies of internal coalition dynamics) and more comparative inquiry to learn why particular EJ coalitions are more effective than others. See the sidebar titled Summary of Lessons from Coalitions.

EJ activists have also forged savvy alliances with another key formal institution: academia. These academic ties stand in contrast to many health activists who regard universities with suspicion, given historical tensions over real estate expansion, exploitative medical experimentation, and general exclusion from institutional fruits (5, 10, 11, 29).

EJ activists have strategically deployed and sought out scientific knowledge, even while sometimes criticizing its narrowness or production. In many cases, EJ activists have expressed discontent with conventional studies that they believe downplay risk because of their myopic scope or how they have been conducted (113). At the same time, to substantiate their intuitive and experiential senses of environmental burdens, they have sought out the technical expertise—and credibility—of academic researchers, working with them to generate better data on environmental health risks, which can then be invoked in political demands (14, 113). Such academic partnerships have become more institutionalized alongside the broader advent of community-based participatory research (CBPR). In its most ideal form, CBPR allows EJ activists to adopt cue-giving roles, not simply to act as consumers or subjects of research that academic experts create. EJ activists point scientists to the very problems to be studied, including ones that experts might have missed; shape many aspects of study design; and question the adequacy of prevailing paradigms in research, whether risk management or narrow conceptions of point sources of pollution (e.g., a focus on indoor air quality in discussions of respiratory disease). Studies conducted with CBPR techniques have analyzed everything from the spatial distribution of risks to community frustrations and needs (4, 14, 15, 33, 80, 85–87).

Methodologically, many instruments, such as survey and interview items or air quality measuring instruments, have been created, validated, and modified in conjunction with community partners themselves. Community members have also shaped sampling. In one CBPR study conducted in a Northern California community near an oil refinery, EJ activists encouraged researchers to widen the spectrum of toxic substances measured so as to ensure that oil refinery emissions would be more fully accounted for (4, 31). Residents also assist, too, with recruitment and actual data collection, serving as trusted conduits between university researchers and those who might otherwise be less inclined to respond to researcher requests for information or involvement. For example, one study in Pittsburgh’s East End, conducted between local organizations and university researchers, employed teams of neighborhood residents to stimulate awareness of EJ issues and encourage residents to outfit bicycles with air quality monitoring instruments (104). Another compelling method is the use of PhotoVoice, which allows participants to take photographs and narrate audiovisual impressions of a local “riskscape,” often as they are also assisting with more traditional data collection (1, 7, 13, 21, 43). For some projects, an additional useful template might be exploratory mixed-methods designs, whereby qualitative inquiry with community members yields insights that inform the design of more traditional quantitative instruments. Surprisingly, though, EJ research on the whole has not adopted formal mixed-methods designs as frequently as one might expect (38).

A final innovation of CBPR EJ studies is their very purpose. Almost all studies, strikingly so, proceed with an explicit desire to impact environmental health policy. They reject simply generating data for its own sake, however provocative or indicting. While exact policy goals may or may not be fully formed at the outset of research, the translation of findings into real-world practical ends is frequently included in the research design, not left as something for others to pursue from basic science findings. One notable project, conducted in Los Angeles, hoped to craft policies that would reduce pollution from the area’s two adjacent ports, the largest shipping complex in the United States, and delay further expansion until EJ ramifications were more clearly studied (45). Another endeavor, carried out in Old Town National City, California (near San Diego), had community members analyze pros and cons of various policy possibilities, which resulted in pursuit of an amortization ordinance that would allow the city government to phase out old sources of emission previously grandfathered and exempted from existing zoning. Advocacy training was built into the research design and included instruction for residents on how to diagram key stakeholders and give public presentations before the city council (86).

CBPR accounts report considerable complementarity between activists and academics, but there exist internal tensions of such collaborations, some explicitly named and some not. Intrinsic power differentials exist between academic partners and activists (27, 36, 90, 125). However equal a partnership may seem, it is the academic partner who typically holds funding and on whose goodwill EJ activists must ultimately rely. Then there is the degree to which more institutionalized alliances with academia mollify the more confrontational and insurgent character of EJ activism, particularly the initial extra-institutional grassroots phase. Put another way, do academic institutions function as safety valves that rechannel political energy into safer venues, including the production of scientific articles? Beyond CBPR itself, some more critical commentators have questioned the inside-out strategy and public policy as an end goal, arguing that they necessitate compromises with the state, which they believe is often complicit in helping bring about the very environmental injustice to be combated (98, 114).

## WORKING IN THE COURTS AND AGENCIES

Courts are another pillar of the EJ inside-outside strategy. EJ organizations and individuals have sued federal agencies for actions—typically related to permitting or environmental assessments and impact statements—that might result in disparate environmental health burdens, which activists characterize as a civil rights or equal protection violation. Less often, agencies have also sued polluters.

The record of EJ litigation is sobering. The theoretical potential of merging civil rights and environmental law has not led to many actual favorable outcomes in the courts. One impediment is the distinction between showing disparate impact itself and showing it but then demonstrating intent and direct discrimination. Scholars have analyzed case law surrounding Section 601 of the Civil Rights Act, which requires any government agency receiving federal funds, such as the Environmental Protection Agency (EPA) and state-level analogs, to adhere to antidiscrimination law. Cases that have made their way to federal courts (up to and through the US Supreme Court) have interpreted Section 601 and affirmed the need for plaintiffs to demonstrate intent, a standard which two scholars have characterized as narrow in that “it ignores larger social forces that may be discriminatory and therefore contribute to environmental injustice” (50, p. 215).

A second impediment is procedural and stems largely from *Alexander v. Sandoval*, a 2001 US Supreme Court case surrounding Section 602, which gives agencies the power to write rules and regulations that uphold Section 601. In *Alexander*, the majority ruled that in cases of disparate impact, the law did not grant a “private right of action” for plaintiffs to sue agencies they believed were noncompliant. These developments—affirmation of an intent standard and the *Alexander* interpretation of Section 602—have hampered successful EJ litigation, at least at the federal level. Similar problems have stymied a similar strategy: invocation of the equal protection clause (the Fourteenth Amendment). Several EJ cases in federal courts have found that plaintiffs inadequately proved “discriminatory intent,” not just disparate environmental impact (50, 55).

Another legal tactic rests on the National Environmental Protection Act (NEPA) and the so-called “hard look doctrine,” which requires government agencies to examine methodologically the impacts of their actions via environmental assessments (EA) and environmental impact statements (EIS) (82). With NEPA, EJ activists have sought to compel agencies to incorporate EJ principles in decision-making. They have been buttressed by Executive Order 12898 (in 1994) and the mandates it theoretically imposes on federal agencies (50, 121). Scholars studying these efforts report mixed results. As with post-*Alexander* Title VI EJ cases, it is generally difficult for plaintiffs to demonstrate that they have the legal standing to sue agencies for ignoring EJ principles spelled out in Executive Order 12898. On the other hand, some federal appeals courts have heard NEPA cases that invoke the Administrative Procedure Act and its ban on “arbitrary and capricious” actions, which plaintiffs have argued include nonconsideration of disparate environmental impacts. Overall, though, NEPA successes, as with Title VI and equal protection ones, are few and far between. Litigation is also frequently mired in arguments over calculations in EAs and EISs, such as highly technical disputes over case comparisons or statistical sampling. This haggling can result in decisions hinging more on methodological than on substantive grounds (55).

State-level actions need to be studied more systematically, as many states have passed EJ-influenced laws. Although appeals do eventually reach federal courts, state-level action may be a way to transcend the considerable impediments posed by invoking federal law. At the state level, some attorneys general have been proactive at filing lawsuits against polluters, thus taking some of the onus off EJ activists themselves (55). And when such cases are appealed and sent to federal courts, the latter have generally upheld the right of states to pass and enforce such statutes, a continuation of long-standing federalist traditions in environmental and public health law (48). It is less clear, however, how prevalent EJ statutes are across the country, what their purview is, and how often and successful litigation is. This is an area for more systematic study, as is more understanding of how state-level EJ regulatory actions are catalyzed by developments at regional and local levels of governance, which defer considerable environmental governance to state-level agencies.

Favorable decisions, however, are not the only purpose that lawsuits can serve. Even if court cases do not always end in an ideal outcome, litigation can still draw public attention to both a particular dispute and EJ more generally (55). Here, the example of environmental tort litigation over toxic substances—most famously tobacco, but also lead, asbestos, polychlorinated biphenyls (PCBs), and others—may be instructive. Whatever the outcome, litigation can serve as a rallying point for EJ activists, while data revealed in the cases can provide important public education (106).

Still, if litigation ultimately does not produce results with the frequency that EJ activists might want, what of agency actions themselves? Here, the evidence also remains mixed. Rather than sue an agency, EJ activists can submit Title VI complaints to the EPA’s own Office of Civil Rights or its External Civil Rights Compliance Office. But many analyses have found that these EPA divisions reject or dismiss the overwhelming majority of complaints while exceeding the legal three-week time limit to make a determination. One EPA-commissioned high-profile assessment by Deloitte, for example, found that the Office of Civil Rights responded on time in only 6% of cases, with some complaints submitted as far back as a decade (39). Another more recent assessment from the US Commission on Civil Rights found that the EPA, between 1993 and 2014, rejected or dismissed 236 complaints, referred or resolved 27, and accepted only 14 (120). The report documented many rejections over procedure, such as exceeding a time limit to file a complaint, even though the EPA had the power to waive it. These analyses, however, were not conducted by academics, and they highlight the need for more inquiry about the EPA’s Title VI complaint process, its strengths and inadequacies, whether resource deficiencies are responsible for the glacial pace of response to complaints, and the extent to which rejections or dismissals occur on procedural versus substantive grounds. We also need to understand state-level agency action; many EJ activists have focused their attention there because of frustration with federal agencies. One model project has compared the EPA and several state-level agencies using both legal and policy analysis along with interviews and participant observation of agency employees (54). More work is also needed on the degree to which states enforce federal laws, which prior research suggests has been limited (71).

It is important to situate EJ litigation within ongoing transformations in administrative law, which governs the powers and limitations of executive agencies, including those responsible for environmental protection. Although courts already had tended toward a strong intent standard, the *Alexander* decision further hampered EJ activists’ attempts to hold agencies accountable in the courtroom, to say nothing of antidiscrimination law generally (23). These developments should be seen as part of long-standing efforts by conservatives to weaken the abilities of federal regulatory agencies that comprise what they call the “administrative state.” A number of cases will soon appear before federal appeals courts and the US Supreme Court that attempt to challenge the power of regulatory agencies, including the precedent set by *Chevron U.S.A., Inc. v. Natural Resources Defense Council, Inc.*, a 1984 US Supreme Court case that granted agencies considerable latitude in interpreting statutes and crafting rules to implement them, so-called Chevron deference (83). One possible harbinger is *West Virginia v. EPA*, the 2022 US Supreme Court decision, which declared former President Barack Obama’s Clean Power Plan an instance of EPA agency overreach. The Court specifically assessed “generation shifting,” which would have required coal power plants to move to more renewable, less-carbon-emitting forms of energy production. The majority decision was anchored in the so-called “major questions” doctrine, which holds that for agency actions of major “economic and political significance,” explicit statutory authority is required. EJ activists have already seen their ability to sue agencies curtailed and a higher burden of proof to establish intent imposed. Now, agencies themselves stand to have their powers clipped, however unsatisfactorily they have exercised them in the eyes of EJ activists. In the coming decade, there is a need for more scholarship probing EJ and the role of courts in what will likely be a very altered federal judicial landscape.

## EJ AND SCIENTIFIC FINDINGS

Several scientific studies explicitly note the influence of EJ—the CBPR EJ research, for example—while others owe an obvious debt to questions that EJ activists have raised. Either way, the scholarship is a testament to the impact that EJ has had on knowledge production itself.

### Neighborhood Characteristics and Spatial Distribution of Emissions

EJ-influenced science has heavily focused on air emissions from industrial sources, waste plants, and automotive emissions. Consequently, the most common health outcome of interest has been the differential incidence of respiratory and pulmonary conditions. There has been, at least in the United States, less attention to differential water (versus air) quality, though this situation may change because of the enormous public attention surrounding the ongoing Flint, Michigan water crisis, first exposed in 2016, which demonstrated that a switch in water sources led to elevated blood lead levels in Flint residents (52).

More recent research has continued along these lines, though there is more focus on levels of emissions—particularly NO_2_, PM_10_, and especially, PM_2.5_—than on the sources from which they emerge. This may be because detailed data sets for these compounds are available from the EPA, especially its Air Quality System and its Toxics Release Inventory. Researchers have also exploited state-level emissions data, which often contain information on particular sources and emissions attributed to them. Some of the most fruitful studies continue to explore links between higher exposures (to either hazardous emissions or proximity to sources) and residential segregation by race and socioeconomic status (SES), particularly median household income and educational attainment (12, 28, 65, 72, 76, 116, 127, 128). In general, and in most locales, nonwhite populations were more exposed than whites, though in almost all instances, Black populations had the most exposure, followed typically by Latinx and Asian populations, respectively. That said, one study found a particularly high predictive effect when the Latinx population was higher than 60% (65, 76). A strong effect also holds for SES; lower-SES neighborhoods consistently exhibit disproportionate exposure.

Researchers have identified various neighborhood characteristics and their influence. They have found that areas with higher housing value and a higher percentage of owner-occupied housing have lower routine emissions and excess emissions. A more curious characteristic is density. One study found that an increase in density was associated with a lower probability of emissions (76). But because high density can be found in both highly segregated and nonaffluent areas—and the opposite—future studies situating findings in the specific social history of a place may better explain such results and highlight unequally distributed burdens on a more granular level. Another compelling finding concerns the intertwining of SES and race and finds that “racialized poverty segregation”—defined as a racially segregated neighborhood in a more economically segregated county—was associated with a higher likelihood of pollution sources (3). This finding follows a growing literature on the impacts of economic segregation, not just across but within racial groups (101, 102).

Another line of inquiry takes a longer-run view on residential segregation and air pollution. One study analyzed data from 1981 to 2016, ranking tracts each year by level of PM_2.5_ exposure. It found that exposure inequalities across census tracts had decreased but still existed by the end of the period. Moreover, the tail ends of the pollution distribution remained largely the same, with the most polluted tracts in 1981 remaining the most polluted ones in 2016 and vice versa. The role of racial and SES composition was less clear cut, though higher population, a greater percentage of whites, higher SES, and lower Hispanic percentage tended to alter tracts’ pollution rankings. Still, the findings suggest that while active neighborhood transformation occurred between the two poles, neighborhoods at the extreme ends saw little change in their protection from or vulnerability to PM_2.5_ exposure (32). Another study examined the period from 1990 to 2009 using not just PM_2.5_ but also NO_2_ and PM_10_ and found similar trends of overall diminished exposure alongside enduring inequalities between white and Black and Latinx exposure (73). These macrolevel, national long-run studies suggest, in short, that air pollution control has been generally successful but in highly uneven ways.

### Institutions

Other scholarship has examined specific institutions. Primary schools are of particular interest because they concentrate large numbers of children in a specific place for prolonged periods of time and thus make them vulnerable to potential exposure. One study classified schools according to how much they were exposed to airborne neurotoxicants. Those in high-risk schools were more likely to qualify for subsidized meals and to be Latinx, Black, or Asian. Moreover, these schools clustered spatially, with some EPA regions having a far higher proportion of schools deemed high-risk than others; Region 2 (New York, New Jersey) had the highest in this case (49). Other studies have systematically examined the relationship of schools to roadways and academic performance. One analysis conducted in Michigan found that more proximity to freeways was associated with a larger percentage of students failing to meet benchmarks, with a larger portion of Black, Latinx, and low-SES students attending such schools. Apart from educational outcomes, freeways were also associated with undesirable respiratory health outcomes, especially asthma (74). The strength of research on schools is that it focuses on places where people spend significant amounts of time other than their homes. Another underdeveloped site for EJ analyses is the workplace, particularly those with respiratory health hazards and large percentages of low-income or minority workers.

### Risk Perception

Another body of work examines not just pollution itself but risk perception. It identifies different varieties of misalignment between perceived and actual pollution and between perceived pollution and concern that one then shows about the problem (30, 69, 100). One study found that living in or closer to areas with greater minority populations led survey respondents to believe that the pollution problem was worse, regardless of whether that turned out to be true or not, leading to neighborhood stigmatization (69). Although the patterns in these studies are not consistent—and may well be place-bound and not widely generalizable—they carry many implications for EJ activists. Low risk perception and concern, especially in areas with objectively high pollution levels, can make rallying support difficult. On the other hand, reflexive stigmatization of neighborhoods stemming from perceptions of environmental health risk may lower broader public support for EJ measures if the public perceives them as helping only stigmatized groups. This latter phenomenon raises the broader question of how much EJ activists should accentuate a more explicit racial equity frame. Two recent studies (53, 111) found that when study participants were shown vignettes on racial disparities in COVID-19 incidence and deaths, their support for mitigation measures dropped. This response reflected a long-standing tradition in the history of American social welfare, where support for policies dropped if the public believed most beneficiaries were racial minorities, even if that was not true.

Relatedly, two recent ethnographic studies examined why residents of areas with high pollution or concentrated environmental health risk hold ambivalent, sometimes outright negative, views toward mitigation or regulation (59, 64). This finding suggests that conveying dangers to vulnerable communities may need improvement—perhaps in the message itself, who delivers it, or the medium used—and that EJ might draw on the literature in risk communication (47). This is especially true when it comes to toxic substances whose effects may be subclinical and less overt (versus something more viscerally obvious, such as diesel bus fumes). At the same time, the risk communication literature can be fruitfully challenged by EJ, given the former’s focus on emergencies and episodes—natural disasters, pandemics—rather than routinized day-to-day health burdens.

### New Sources of Environmental Injustice: Extractive Sites and Heat Burdens

Investigators have increasingly turned to studying different kinds of pollution sources, a shift in interest almost surely linked to public attention around fossil fuels and climate change. One case is extractive sites. Several studies have examined the role of hydraulic fracturing and a host of outcomes, including birth complications, asthma, headaches, sinus problems, and mental health ailments (22, 99, 117, 118). Similarly, others have examined the health hazards posed by surface mining and mountaintop removal (2, 42, 56). As with hydraulic fracturing, much coal extraction has occurred in low-SES, higher-poverty areas (the Marcellus Shale region, Central Appalachia), including many that are overwhelmingly white, even as the energy produced by those processes is enjoyed by the rest of the country.

The unequal impacts of heat have also attracted increasing attention. Several studies have examined urban heat islands: the tendency of some parts of an urbanized area to have elevated temperatures because of a dense built environment (62, 77, 123). National studies using tract-level temperature data have consistently found that minority and low-income neighborhoods were more likely to experience elevated temperatures and extreme heat. The mechanisms behind this finding are less clear, though researchers have speculated that vegetation and open green space have a strong mitigation effect. Others have examined the spatial distribution of mitigation strategies, including vegetation, green roofs, and surface reflectivity and found occasional unevenness. For example, one study of three metropolitan areas—Phoenix, Atlanta, and Philadelphia—found that the benefits of higher surface reflectivity, as measured by estimated avoided mortality, were 27% higher in majority white census tracts (123). By contrast, in Philadelphia, there were no racial differences in various mitigation strategies. When it came to income, Phoenix exhibited no differences in mitigation impact. But Philadelphia did, with the advantages of some strategies, such as vegetation and surface reflectivity, not surfacing in poorer areas (123). Such results suggest that some municipalities have been more effective at equitably distributing risk mitigation than have others, and further case studies examining policy implementation would be instructive.

Heat studies also point to another characteristic of more recent scholarship. There is a focal shift from external threats to positive assets that counter environmental health harms, which is evident in work examining access to green space, parks, and tree coverage. Authors generally find income and race differentials, though many also highlight city-to-city variation in patterns (8, 9, 35, 44, 77, 91, 105, 108). A corollary of that work examines, often critically, policies and programs to catalyze such protective resources. Some work has found that tree-planting programs, for example, end up exacerbating environmental health inequity because of the greater likelihood of affluent individuals participating in them, as happened with a voluntary Portland program (40). Others have explored more ambitious citywide programs, such as New York City’s and Los Angeles’s high-profile MillionTreesNYC and MillionTreesLA initiatives. One study found that in New York City, additional trees tended to be planted in areas with higher canopy cover and fewer nonwhite residents. In Los Angeles, the study found more promising results but faulted the initiative for collecting less-than-thorough data that would have enabled a fuller evaluation (46).

### Policy Context

Finally, a growing number of studies place findings on various outcomes in a larger policy context. In the past few years, much of this work has centered around redlining. Scholars have associated entrenched residential segregation and a host of environmental health hazards with neighborhoods that were historically redlined by government agencies such as the Home Owners Loan Corporation (HOLC). This research has largely been catalyzed by digitized HOLC maps, which allow for much easier analysis in geographic information systems packages (75, 92). Other work has examined such legislation as the Clean Air Act and its long-run impact on exposures, in one study’s case, PM_2.5_ (34).

## FUTURE DIRECTIONS

The EJ literature is sprawling but energetic, with many potential future directions. This concluding section offers four generative areas for the field to pursue.

### Coalitions

Coalitions are a core component of EJ activism, and scholars should continue highlighting which stakeholders and organizations forge alliances around EJ. Two additional players that will likely attract more sustained attention are immigrant and Indigenous rights groups, many of whom have been adding EJ to their organizational missions (24, 68). While both have long been active in EJ causes, there has been more pronounced public visibility and, for some, renewed commitment given events of the past decade: anti-immigrant, nativist federal policies in the United States and the Standing Rock protests over the construction of the Dakota Access Pipeline, which ran near tribal lands and threatened water sources (124). This higher visibility follows criticism of Native Americans’ absence at some EJ convenings and a lack of attention to environmental health issues on tribal lands. Labor is a third constituency to note, given a string of recent state-level victories for farmworkers’ rights, such as New York State’s Farm Laborers Fair Labor Practices Act, which allows agricultural workers to collectively bargain and join unions without retaliation.

### Fragmentation

EJ literature is strongly bifurcated between works that center on low-SES Black, Indigenous, Latinx, and Asian neighborhoods and those that study low-SES predominantly white areas, typically those bearing the brunt of new forms of energy extraction. There should be more efforts by researchers to bring these two bodies of scholarship into conversation with each other. For scholars, EJ activism from both types of regions is ripe for comparative inquiry that can elucidate the relative success or failure of the other. And for EJ activists, drawing links may also be a form of coalition building, given the costs associated with industrial growth and energy extraction incurred by both types of places.

The most urgent fissure is between EJ and climate justice. Whereas traditional EJ activism and scholarship tend to be bound by locality or nation-state, climate justice work focuses on global disparities in impacts across regions or nation-states (70, 79, 95, 107, 114). More recently, however, this distinction has begun to blur, as EJ activists consider the larger-scale potential of their work beyond the local scale. Such multilevel thinking also partially addresses an enduring limitation of local EJ activism, where one local victory simply results in a hazard moving elsewhere or where scattered local victories do not result in more large-scale transformation of conditions that led to these hazards in the first place. Climate justice helps EJ consider interregional interconnectivity—both within and across national borders—which much of the literature still lacks.

The Inflation Reduction Act, passed in 2022, offers encouraging signs that EJ and climate justice may be converging. Most attention has focused on the bill’s climate initiatives, which spearhead development of renewable energy infrastructure and technology, plus incentivize consumer adoption of electric vehicles and other clean-energy products. However, EJ provisions have been noticed less, including $2.8 billion in block grants to community organizations engaging in EJ work, to be supervised by the EPA’s newly created Office of Environmental Justice and External Civil Rights. The Act has also created a $27 billion Greenhouse Gas Reduction Fund, $15 billion of which is aimed at spearheading climate infrastructure in “low-income and disadvantaged communities.” Other allocations include improving water access for “disadvantaged communities” ($550 million) and creating climate resilience projects for Native American and Native Hawaiian lands (more than $400 million) (41). How exactly the funds are distributed remains to be seen, and monitoring actual implementation will be an important task for EJ researchers. But the inclusion of EJ provisions in a climate change bill recognizes that the fights over disproportionate burdens and unsustainable carbon emissions are increasingly the same.

### Processual/Historical Analysis

Generations of quantitative EJ studies have identified patterns of source, exposure, and mitigation maldistribution. They have been less effective, however, at explaining the public policy decisions, regulations and rules, demographic transformations, migration patterns, political arrangements, and corporate behavior that have led to those patterns. Put another way, EJ studies are often better at rendering what a spatial configuration looks like and analyzing what its downstream health consequences might be than they are at explaining the upstream forces that created them. While qualitative studies more effectively capture these elements, they tend to have a limited temporal scope—typically the time period during which a researcher is conducting participant observation—and limited historical exposition. There is thus a need for more long-run, processual analysis in the vein of historical accounts of cities that can explain the decades-long origins of their contemporary problems (112).

This scholarship, however, is sparse and should be more commonplace and incorporated into field debates writ large. One model account is Hurley’s (63) history of Gary, Indiana, and the contradictions inherent in simultaneous post-WWII dependence on the steel industry for employment and the environmental and occupational health consequences associated with the location of steel facilities near working-class and Black neighborhoods. Spears (109) documents the contamination of Anniston, Alabama, over a decades-long period with PCBs manufactured at a Monsanto plant, which resulted in high PCB blood levels for Anniston residents. She also analyzes a groundswell of EJ activism that culminated in civil suits attempting to hold Monsanto to account. More recently, Rector has offered a sweeping account of various struggles in Detroit in the twentieth century. He analyzes the fall of a once vibrant organized labor-environment coalition, then turns to the city’s struggling fiscal condition and the dubious maneuvers that elected officials took to address municipal debt, such as selling a waste incinerator that polluted disproportionately one of the city’s Black neighborhoods (103). Rector’s study offers a political-economic backdrop to the cost-cutting maneuvers that led to the city’s infamous water crisis. All these studies also extend our understanding of EJ chronology, moving many decades before the common start date in the late 1970s and early 1980s in the American South (110).

Historical approaches may also shed light on the relationship between race and class, a perennial debate that has bedeviled EJ activism and scholarship. The question is often addressed quantitatively, with studies purporting to show the relative importance of one by controlling for the other. Processual analysis instead shows how political-economic forces, institutional context, and racial animus often combine to produce racially disparate environmental health risks. A key takeaway of these accounts, though, is that the race–class dynamic varies depending on time period and place and cannot be characterized by way of a general abstraction.

Deep excavation of the past can further explain strong associations that have been found with HOLC boundaries and environmental health hazards. An existing literature situates HOLC in larger redlining regimes that involved a much wider constellation of actors, including private real estate interests (pre- and post-HOLC) and the Federal Housing Authority. Incorporating this approach would evolve the HOLC and environmental health work by identifying which institutions and actors were embedded in redlining, how they contributed to residential segregation at different moments and in different places, and, later, the patterning of environmental health hazards (58, 84, 112, 126).

Because such accounts use data (archival sources) and take forms (long-form narrative) that are not often used and adopted in conventional public health research, they are rarely well-subsidized by traditional sources of research support and face some of the same funding obstacles experienced in CBPR work.

### Inserting EJ Optics

Finally, EJ scholars should insert EJ concerns into other fields that have identified environmental health contributions to various health outcomes. One example is the growing scholarship on maternal-child health disparities, which finds that environmental health exposures over the life course can result in birth complications (93). This research, however, is not always cognizant of EJ framing that might reveal the social forces responsible for the unequal distribution of such exposures. This is in spite of calls, more than a decade ago, to find more commonalities across domains (89). Cross-cutting of this kind may also help EJ activists forge coalitions with nonenvironmental groups.

## CONCLUSION

The scope of EJ makes the task of capturing its general shifts and promising future directions a daunting one. That sprawl, however, should be read as a sign of vibrancy. Within public health, there are few other fields that bring together activists, scholars, and practitioners; that are both analytic and practice oriented; and that seek not only to understand the world but to actively change it for the better. But a radically changing legal environment and the climate crisis necessitate adaptation and a constant rethinking of what has and has not worked.

## SUMMARY OF LESSONS FROM COALITIONS

- EJ activism is tactically diverse.
- EJ activists frequently employ an inside-outside strategy, both agitating outside of formal institutions (e.g., courts, legislatures, and universities) and also engaging directly with them.
- EJ coalitions often cut across sectors and include nonenvironmental organizations and actors. They also include different organizations fighting for EJ principles.
- Case studies of EJ coalitions have uncovered frictions among coalition members, especially when some perceive others as acquiescing to an acceptable level of environmental health burden.
- Alliances between EJ activists and universities are one particularly successful EJ coalition. This lay-expert cooperation has produced novel scientific insights on the distribution of environmental health risks. It has in turn aided EJ activists’ credibility as activists make claims.
- Some critics, past and present, have questioned an inside-outside strategy, arguing that it may necessitate dilution of demands and conciliation with governments and other formal institutions.

## SUMMARY POINTS

1. Environmental justice (EJ) is many things: a multidisciplinary, multimethod field of academic inquiry; a current of activism that has existed for decades; and an administrative imperative adopted by government agencies and incorporated into legislation.
2. EJ activism is often composed of coalitions consisting of multiple actors across sectors and those united around a common goal. These coalitions, however, are not without internal tensions that sometimes play out over time.
3. EJ activism has spawned fruitful cooperation between academic researchers and lay EJ activists.
4. Community-based participatory research (CBPR) has been one particularly fruitful approach in EJ research and has employed novel data collection practices and study designs that have yielded important insights on environmental health hazards faced by local neighborhoods.
5. EJ attempts to fuse together the powers of civil rights law and environmental law, but results from litigation and from formal agency complaint processes have been decidedly mixed because of a number of factors. These factors include an increasingly conservative judiciary suspicious of federal agency overreach, stricter application of intent standards with regard to racially disparate outcomes, and bottlenecking and inefficiency at federal agencies themselves.
6. Neighborhood characteristics, especially higher residential segregation and lower SES levels, are closely associated with higher pollution levels.
7. New work is examining not just spatial location of hazards but also the ways that hazards are perceived by vulnerable communities. It is also expanding beyond just emissions and examining the maldistribution of tree coverage and heat burden.
8. Climate change and EJ activism are increasingly fusing, as exemplified in the strong EJ presence in the recently passed US Inflation Reduction Act.

## Abbreviations

Environmental justice (EJ): the academic study and practice of combatting the disproportionate presence of environmental health risks in areas with high percentages of low-income residents and racial minorities
Executive Order 12898: directed all federal agencies to examine and address the disparate impacts that their activities might have on low-income and minority areas
Inside-outside strategy: a tactic, employed frequently by EJ coalitions, of agitating outside—and sometimes against—formal institutions (e.g., courts, agencies, legislatures, universities) while also working with them at opportune points
Coalitions: political formations common in EJ where different stakeholders unite against a common cause while attempting to balance inherent tensions that might later surface between them
Community-based participatory research (CBPR): academic research conducted with lay nonacademics or community organizations where the latter have substantial input in study design and issues to be researched
Disparate impact: a racially disproportionate outcome (in distribution of environmental health hazards or otherwise) that need not—though can—require explicit intent to occur
Intent standard: an increasingly common legal doctrine whereby EJ advocates seeking redress have had to demonstrate not just a disparate environmental health outcome but direct intent to produce it
Administrative state: the Executive Branch’s federal agencies (e.g., the EPA), whose regulation- and rule-making authority has been under increasing challenge, impeding potential future action on behalf of EJ
*Chevron* deference: a federal legal precedent that gives federal agencies broad latitude in interpreting statute authority and crafting rules and regulations to carry it out
Major questions: a legal doctrine, in tension with *Chevron* deference, which requires federal agency actions with major “economic and political significance” be explicitly permitted by a statute
Climate justice: the growing social movement attempting to compel governmental action for climate change mitigation
Inflation Reduction Act: a landmark 2022 federal law that allocated billions, by some estimates, for EJ activity alongside a broader investment in climate change mitigation

## References

[1] Aber A, Waxman N, Khatib A, Bamfo A, Simon-Ogan D, Wilson S. 2017. Use of photovoice to highlight environmental justice issues: the power of photography in Buzzard Point, Washington, DC. Environ. Justice 10:36–42

[2] Ahern MM, Hendryx M, Conley J, Fedorko E, Ducatman A, Zullig KJ.2011.The association between mountaintop mining and birth defects among live births in central Appalachia, 1996–2003. Environ. Res 111:838–46 21689813 10.1016/j.envres.2011.05.019

[3] Ard K, Smiley K. 2021. Examining the relationship between racialized poverty segregation and hazardous industrial facilities in the U.S. over time. Am. Behav. Sci 66:974–88

[4] Balazs CL, Morello-Frosch R. 2013. The three Rs: how community-based participatory research strengthens the rigor, relevance, and reach of science. Environ. Justice 6:9–16 10.1089/env.2012.0017 PMC3832061 24260590

[5] Baldwin DL. 2021. In the Shadow of the Ivory Tower: How Universities are Plundering Our Cities. New York: Bold Type Books

[6] Bell SE.2013. Our Roots Run Deep as Ironweed: Appalachian Women and the Fight for Environmental Justice. Urbana: Univ. Ill. Press

[7] Bell SE. 2015. Bridging activism and the academy: exposing environmental injustices through the feminist ethnographic method of photovoice. Hum. Ecol. Rev 21:27–58

[8] Bloch SK. 2019. Shade. Places J 10.22269/190423

[9] Boulton C, Dedekorkut-Howes A, Byrne J. 2018. Factors shaping urban greenspace provision: a systematic review of the literature. Landsc. Urban Plan 178:82–101

[10] Boulware LE, Cooper LA, Ratner LE, LaVeist TA, Powe NR. 2003. Race and trust in the health care system. Public Health Rep. 118:358–65 12815085 10.1016/S0033-3549(04)50262-5 PMC1497554

[11] Bradley SM. 2012. Harlem vs. Columbia University: Black Student Power in the Late 1960s. Urbana: Univ. Ill. Press

[12] Brewer M, Kimbro RT, Denney JT, Osiecki KM, Moffett B, Lopez K. 2017. Does neighborhood social and environmental context impact race/ethnic disparities in childhood asthma? Health Place 44:86–93 28219854 10.1016/j.healthplace.2017.01.006 PMC6728803

[13] Brickle MB, Evans-Agnew R. 2017. Photovoice and youth empowerment in environmental justice research: a pilot study examining woodsmoke pollution in a Pacific Northwest community. J. Community Health Nurs 34:89–101 28467206 10.1080/07370016.2017.1304148

[14] Brown P, Mayer B, Zavestoski S, Luebke T, Mandelbaum J, McCormick S. 2003. The health politics of asthma: environmental justice and collective illness experience. Soc. Sci. Med 57:453–64 12791488 10.1016/s0277-9536(02)00375-1

[15] Brown P, Morello-Frosch R, Zavetoski S. 2012. Getting into the field: new approaches to research methods. In Contested Illnesses: Citizens, Science, and Health Social Movements, ed. Brown P, Morello-Frosch R, Zavetoski S, pp. 46–63. Berkeley: Univ. Calif. Press

[16] Brownson RC, Kumanyika SK, Kreuter MW, Haire-Joshu D. 2021. Implementation science should give higher priority to health equity. Implement Sci. 16(1):28 33740999 10.1186/s13012-021-01097-0 PMC7977499

[17] Brulle RJ, Pellow DN. 2006. Environmental justice: human health and environmental inequalities. Annu. Rev. Public Health 27:103–24 16533111 10.1146/annurev.publhealth.27.021405.102124

[18] Bullard RD. 2000. Dumping in Dixie: Race, Class, and Environmental Quality. New York: Routledge. 3rd ed.

[19] Bullard RD. 2015. Environmental justice in the 21^st^ century: Race still matters. Phylon 52:72–94

[20] Bullard RD, Johnson GS. 2000. Environmental justice: grassroots activism and its impact on public policy decision making. J. Soc. Issues 56:555–78

[21] Cannuscio CC, Weiss EE, Fruchtman H, Schroeder J, Weiner J, Asch DA. 2009. Visual epidemiology: photographs as tools for probing street-level etiologies. Soc. Sci. Med 69:553–64 19573966 10.1016/j.socscimed.2009.06.013

[22] Casey JA, Savitz DA, Rasmussen SG, Ogburn EL, Pollak J, 2016. Unconventional natural gas development and birth outcomes in Pennsylvania, USA. Epidemiology 27:163–72 26426945 10.1097/EDE.0000000000000387 PMC4738074

[23] Ceballos CI, Engstrom DF, Ho DE. 2021. Disparate limbo: how administrative law erased antidiscrimination. Yale Law J. 131:370–474

[24] Chandrasekaran PR. 2021. Remaking “the people”: immigrant farmworkers, environmental justice and the rise of environmental populism in California’s San Joaquin Valley. J. Rural Stud 82:595–605 32952285 10.1016/j.jrurstud.2020.08.043 PMC7487076

[25] Checker M. 2001. “Like Nixon coming to China”: finding common ground in a multi-ethnic coalition for environmental justice. Anthropol. Q 74:135–46

[26] Checker M. 2005. Polluted Promises: Environmental Racism and the Search for Justice in a Southern Town. New York: NYU Press

[27] Chen Y-W, Milstein T, Anguiano C, Sandoval J, Knudsen L. 2012. Challenges and benefits of community-based participatory research for environmental justice: a case of collaboratively examining ecocultural struggles. Environ. Commun 6:403–21

[28] Chi GC, Hajat A, Bird CE, Cullen MR, Griffin BA, 2016. Individual and neighborhood socioeconomic status and the association between air pollution and cardiovascular disease. Environ. Health Perspect 124:1840–47 27138533 10.1289/EHP199 PMC5132637

[29] Chowkwanyun M. 2022. All Health Politics Is Local: Community Battles for Medical Care and Environmental Health. Chapel Hill: Univ. N. C. Press

[30] Cisneros R, Brown P, Cameron L, Gaab E, Gonzalez M, 2017. Understanding public views about air quality and air pollution sources in the San Joaquin Valley, California. J. Environ. Public Health 2017:4535142 28469673 10.1155/2017/4535142 PMC5392406

[31] Cohen A, López A, Malloy N, Morello-Frosch R. 2012. Our environment, our health: a community-based participatory environmental health survey in Richmond, CA. Health Educ. Behav 39:198–209 21742947 10.1177/1090198111412591

[32] Colmer J, Hardman I, Shimshack J, Voorheis J. 2020. Disparities in PM_2.5_ air pollution in the United States. Science 369:575–78 32732425 10.1126/science.aaz9353

[33] Corburn J. 2005. Street Science: Community Knowledge and Environmental Health Justice. Cambridge, MA: MIT Press

[34] Currie J, Voorheis J, Walker R. 2020. What caused racial disparities in particulate exposure to fall? New evidence from the Clean Air Act and satellite-based measures of air quality. NBER Work. Pap 26559. https://www.nber.org/system/files/working_papers/w26659/w26659.pdf

[35] Dai D. 2011. Racial/ethnic and socioeconomic disparities in urban green space accessibility: Where to intervene?. Landsc. Urban Plan 102:234–44

[36] Davis LF, Ramírez-Andreotta MD. 2021. Participatory research for environmental justice: a critical interpretive synthesis. Environ. Health Perspect 129:26001 33591210 10.1289/EHP6274 PMC7885999

[37] De Onis CM, Pezzullo PC. 2018. The ethics of embodied engagement: ethnographies of environmental justice. See Ref. 60, pp. 231–40

[38] DeJonckheere M, Lindquist-Grantz R, Toraman S, Haddad K, Vaughn LM. 2018. Intersection of mixed methods and community-based participatory research: a methodological review. J. Mixed Methods Res 13:481–502

[39] Consult Deloitte. 2011. Evaluation of the EPA office of civil rights. Final Rep., Deloitte Consult, Washington, DC. https://archive.epa.gov/epahome/ocr-statement/web/pdf/epa-ocr_20110321_finalreport.pdf

[40] Donovan GH, Mills J. 2014. Environmental justice and factors that influence participation in tree planting programs in Portland, Oregon, U.S. Arboricult. Urban For 40:70–77

[41] Environ. Energy Law Program (Harvard Law School). 2022. Environmental justice (EJ) provisions of the 2022 Inflation Reduction Act. Table, Environ. Energy Law Program, Harvard Univ., Cambridge, UK. http://eelp.law.harvard.edu/wp-content/uploads/EELP-IRA-EJ-Provisions-Table.pdf

[42] Esch L, Hendryx M. 2011. Chronic cardiovascular disease mortality in mountaintop mining areas of central Appalachian states. J. Rural Health 27:350–57 21967378 10.1111/j.1748-0361.2011.00361.x

[43] Evans-Agnew RA, Postma J, Dinglasan-Panlilio J, Yuwen W, Reyes D, 2022. “Is It Good or Bad for the Air?” Latino and Asian Pacific Islander youth-led messaging and action for environmental justice through photovoice. Health Promot. Pract 23:305–16 35285315 10.1177/15248399211045729

[44] Flocks J, Escobedo F, Wade J, Varela S, Wald C. 2011. Environmental justice implications of urban tree cover in Miami-Dade County, Florida. Environ. Justice 4:125–34

[45] Garcia AP, Wallerstein N, Hricko A, Marquez JN, Logan A, 2013. THE (Trade, Health, Environment) Impact Project: a community-based participatory research environmental justice case study. Environ. Justice 6:17–26

[46] Garrison JD. 2021. Environmental justice in theory and practice: measuring the equity outcomes of Los Angeles and New York’s “Million Trees” campaigns. J. Plan. Educ. Res 41:6–17

[47] Glik DC. 2007. Risk communication for public health emergencies. Annu. Rev. Public Health 28:33–54 17222081 10.1146/annurev.publhealth.28.021406.144123

[48] Gostin LO. 2008. Public Health Law: Power, Duty, Restraint. Berkeley: Univ. Calif. Press

[49] Grineski SE, Collins TW. 2018. Geographic and social disparities in exposure to air neurotoxicants at U.S. public schools. Environ. Res 161:580–87 29245126 10.1016/j.envres.2017.11.047 PMC5760180

[50] Gross E, Stretesky P. 2015. Environmental justice in the courts. In Failed Promises: Evaluating the Federal Government’s Response to Environmental Justice, ed. Konisky DM, pp. 205–31. Cambridge, MA: MIT Press

[51] Hall RL, Jacobson PD. 2018. Examining whether the health-in-all-policies approach promotes health equity. Health Aff. 37:364–70 10.1377/hlthaff.2017.1292 29505382

[52] Hanna-Attisha M, LaChance J, Sadler RC, Schnepp AC. 2016. Elevated blood lead levels in children associated with the Flint drinking water crisis: a spatial analysis of risk and public health response. Am. J. Public Health 106:283–90 26691115 10.2105/AJPH.2015.303003 PMC4985856

[53] Harell A, Lieberman E. 2021. How information about race-based health disparities affects policy preferences: evidence from a survey experiment about the COVID-19 pandemic in the United States. Soc. Sci. Med 277:113884 33845391 10.1016/j.socscimed.2021.113884 PMC8635307

[54] Harrison JL. 2019. From the Inside Out: The Fight for Environmental Justice with Government Agencies. Cambridge, UK: MIT Press

[55] Henderson DA, Stroman CAM, Eisert JA. 2021.Environmental justice litigation: few wins,still effective. Natl. Resour. Environ 36:17–21

[56] Hendryx M, Zullig KJ, Luo J. 2020. Impacts of coal use on health. Annu. Rev. Public Health 41:397–415 31913772 10.1146/annurev-publhealth-040119-094104

[57] Hess DJ, Satcher LA. 2019. Conditions for successful environmental justice mobilizations: an analysis of 50 cases. Environ. Politics 28:663–84

[58] Hirsch AR. 1983. Making the Second Ghetto: Race and Housing in Chicago, 1940–1960. Chicago: Univ. Chicago Press

[59] Hochschild AR. 2018. Strangers in Their Own Land: Anger and Mourning on the American Right. New York: New Press

[60] Holifield R, Chakraborty J, Walker G, eds. 2018. The Routledge Handbook of Environmental Justice. New York: Routledge

[61] Hoover E. 2017. The River Is in Us: Fighting Toxics in a Mohawk Community. Minneapolis: Univ. Minneap. Press

[62] Hsu A, Sheriff G, Chakraborty T, Manya D. 2021. Disproportionate exposure to urban heat island intensity across major US cities. Nat. Commun 12:2721 34035248 10.1038/s41467-021-22799-5 PMC8149665

[63] Hurley A. 1996. Environmental Inequalities: Class, Race, and Industrial Pollution in Gary, Indiana, 1945–1980. Chapel Hill: Univ. N. C. Press

[64] Jerolmack C. 2021. Up to Heaven and Down to Hell: Fracking, Freedom, and Community in an American Town. Princeton, NJ: Princeton Univ. Press

[65] Jones MR, Diez-Roux AV, Hajat A, Kershaw KN, O’Neill MS, 2014. Race/ethnicity, residential segregation, and exposure to ambient air pollution: the Multi-Ethnic Study of Atherosclerosis (MESA). Am. J. Public Health 104:2130–37 25211756 10.2105/AJPH.2014.302135 PMC4202969

[66] Karner A, Eiseinger D, Bai S, Niemeier D. 2009. Mitigating diesel truck impacts in environmental justice communities: transportation planning and air quality in Barrio Logan, San Diego, California. Transp. Res. Record 2125:1–8

[67] Karner A, London J, Rowangould D, Manaugh K. 2020. From transportation equity to transportation justice: within, through, and beyond the state. J. Plan. Lit 35:440–59

[68] Kim NY. 2021. Refusing Death: Immigrant Women and the Fight for Environmental Justice in LA. Stanford, CA: Stanford Univ. Press

[69] King KE. 2015. Chicago residents’ perceptions of air quality: objective pollution, the built environment, and neighborhood stigma theory. Popul. Environ 37:1–21 26527847 10.1007/s11111-014-0228-x PMC4627697

[70] Klein N. 2015. This Changes Everything: Capitalism vs. The Climate. New York: Simon & Schuster

[71] Konisky DM. 2009. The limited effects of federal environmental justice policy on state enforcement. Policy Stud. J 37:475–96

[72] Kranjac AW, Kimbro RT, Denney JT, Osiecki KM, Moffett BS, Lopez KN. 2017. Comprehensive neighborhood portraits and child asthma disparities. Matern. Child Health J 21:1552–62 28181157 10.1007/s10995-017-2286-z PMC6707800

[73] Kravitz-Wirtz N, Crowder K, Hajat A, Sass V. 2016. The long-term dynamics of racial/ethnic inequality in neighborhood and pollution exposure, 1990–2009. Du Bois Rev. 13:237–59 28989341 10.1017/S1742058X16000205 PMC5628744

[74] Kweon B-S, Mohai P, Lee S, Sametshaw AM. 2018. Proximity of public schools to major highways and industrial facilities, and students’ school performance and health hazards. Environ. Plan. B Urban Anal. City Sci 45:312–29

[75] Lane HM, Morello-Frosch R, Marshall JD, Apte JS. 2022. Historical redlining is associated with present-day air pollution disparities in U.S. cities. Environ. Sci. Technol. Lett 12:345–50 10.1021/acs.estlett.1c01012 PMC9009174 35434171

[76] Li Z, Konisky DM, Zirogiannis N. 2019. Racial, ethnic, and income disparities in air pollution: a study of excess emissions in Texas. PLOS ONE 14:e0220696 31374099 10.1371/journal.pone.0220696 PMC6677308

[77] Lino Sanchez L, Reames TG. 2019. Cooling Detroit: a socio-spatial analysis of equity in green roofs as an urban heat island mitigation strategy. Urban For. Urban Green 44:126331

[78] Marcantonio RA, Golub A, Karner A, Nelson L. 2017. Confronting inequality in metropolitan regions: realizing the promise of civil rights and environmental justice in metropolitan transportation planning. Fordham Urban Law J. 44:1017–78

[79] Martinez-Alier J, Temper L, Del Bene Scheidel A. 2016. Is there a global environmental justice movement? J. Peasant Stud 43:731–55

[80] Masri S, LeBrón AMW, Logue MD, Valencia E, Ruiz A, 2021. Risk assessment of soil heavy metal contamination at the census tract level in the city of Santa Ana, CA: implications for health and environmental justice. Environ. Sci. Process. Impacts 23:812–30 33954329 10.1039/d1em00007a PMC8224146

[81] McCarthy D. 2004. Environmental justice grantmaking: elites and activists collaborate to transform philanthropy. Sociol. Inq 74:250–70

[82] Mercola M. 2020. The hard look doctrine: how disparate impact theory can inform agencies on proper implementation of NEPA regulations. J. Law Policy 28:318–55

[83] Metzger GE. 2017. Foreward: 1930s redux: the administrative state under siege. Harv. Law Rev 131:2–95

[84] Michney TM, Winling LC. 2020. New perspectives on New Deal housing policy: explicating and mapping HOLC loans to African Americans. J. Urban History 46:150–80

[85] Minkler M. 2010. Linking science and policy through community-based participatory research to study and address health disparities. Am. J. Public Health 100:S81–87 20147694 10.2105/AJPH.2009.165720 PMC2837446

[86] Minkler M, Garcia AP, Williams J, LoPresti T, Lilly J. 2010. Sí se puede: using participatory research to promote environmental justice in a Latino community in San Diego, California. J. Urban Health 87:796–812 20683782 10.1007/s11524-010-9490-0 PMC2937121

[87] Minkler M, Vásquez VB, Shepard P. 2006. Promoting environmental health policy through community based participatory research: a case study from Harlem, New York. J. Urban Health 83:101–10 16736358 10.1007/s11524-005-9010-9 PMC2258322

[88] Mohai P, Pellow DN, Timmons Roberts J. 2009. Environmental justice. Annu. Rev. Environ. Resour 34:405–30

[89] Morello-Frosch R, Shenassa ED. 2006. The environmental “riskscape” and social inequality: implications for explaining maternal and child health disparities. Environ. Health Perspect 114:1150–53 16882517 10.1289/ehp.8930 PMC1551987

[90] Muhammad M, Wallerstein N, Susan AL, Avila M, Beloe L, Duran B. 2015. Reflections on researcher identity and power: the impact of positionality on community based participatory research (CBPR) processes and outcomes. Crit. Sociol 41:1045–63 10.1177/0896920513516025 PMC4943756 27429512

[91] Namin S, Xu W, Zhou Y, Beyer K. 2020. The legacy of the Home Owners’ Loan Corporation and the political ecology of urban trees and air pollution in the United States. Soc. Sci. Med 246:112758 31884239 10.1016/j.socscimed.2019.112758

[92] Nardone A, Rudolph KE, Morello-Frosch R, Casey JA. 2021. Redlines and greenspace: the relationship between historical redlining and 2010 greenspace across the United States. Environ. Health Perspect 129:17006 33502254 10.1289/EHP7495 PMC7839347

[93] Padula AM, Monk C, Brennan PA, Borders A, Barrett ES, 2020. A review of maternal prenatal exposures to environmental chemicals and psychosocial stressors—implications for research on perinatal outcomes in the ECHO program. J. Perinatol. 40:10–24 31616048 10.1038/s41372-019-0510-y PMC6957228

[94] Pellow DN. 2002. Garbage Wars: The Struggle for Environmental Justice in Chicago. Cambridge, MA: MIT Press

[95] Pellow DN. 2007. Resisting Global Toxics: Transnational Movements for Environmental Justice. Cambridge, MA: MIT Press

[96] Pellow DN, Brulle RJ. 2005. Power, Justice, and the Environment: A Critical Appraisal of the Environmental Justice Movement. Cambridge, MA: MIT Press

[97] Pulido L, De Lara J. 2018. Reimagining ‘justice’ in environmental justice: radical ecologies, decolonial thought, and the Black radical tradition. Environ. Plan. E Nat. Space 1:76–98

[98] Pulido L, Kohl E, Cotton N-M. 2016. State regulation and environmental justice: the need for strategy reassessment. Capital. Nat. Social 27:12–31

[99] Rasmussen SG, Ogburn EL, McCormack M, Casey JA, Bandeen-Roche K, 2016. Association between unconventional natural gas development in the Marcellus shale and asthma exacerbations. JAMA Intern. Med 176:1334–43 27428612 10.1001/jamainternmed.2016.2436 PMC5424822

[100] Reames TG, Bravo MA. 2019. People, place and pollution: investigating relationships between air quality perceptions, health concerns, exposure, and individual-and area-level characteristics. Environ. Int 122:244–55 30449629 10.1016/j.envint.2018.11.013

[101] Reardon SF, Bischoff K. 2011. Income inequality and income segregation. Am. J. Sociol 116:1092–153 10.1086/657114 21648248

[102] Reardon SF, Bischoff K, Owens A, Townsend JB. 2018. Has income segregation really increased? Bias and bias correction in sample-based segregation estimates. Demography 55:2129–60 30328018 10.1007/s13524-018-0721-4

[103] Rector J. 2022. Toxic Debt: An Environmental Justice History of Detroit. Chapel Hill: Univ. N. C. Press

[104] Rickenbacker H, Brown F, Bilec M. 2019. Creating environmental consciousness in underserved communities: implementation and outcomes of community-based environmental justice and air pollution research. Sustain. Cities Soc 47:101473

[105] Rigolon A. 2019.Nonprofits and park equity in Los Angeles: a promising way forward for environmental justice. Urban Geogr. 40:984–1009

[106] Rosner D. 2009. Trials and tribulations: what happens when historians enter the courtroom. Law Contemp. Probl 72:137–58

[107] Schlosberg D, Collins LB. 2014. From environmental to climate justice: climate change and the discourse of environmental justice. WIREs Clim. Change 5:359–74

[108] Schwarz K, Fragkias M, Boone CG, Zhou W, McHale M, 2015. Trees grow on money: urban tree canopy cover and environmental justice. PLOS ONE 10:e0122051 25830303 10.1371/journal.pone.0122051 PMC4382324

[109] Spears EG. 2014. Baptized in PCBs: Race, Pollution, and Justice in an All-American Town. Chapel Hill: Univ. N. C. Press

[110] Spears EG. 2019. Rethinking the American Environmental Movement Post-1945. New York: Routledge

[111] Stephens-Dougan L. 2022. White Americans’ reactions to racial disparities in COVID-19. Am. Political Sci. Rev 10.1017/S000305542200051X. In press

[112] Sugrue TJ. 1996. The Origins of the Urban Crisis: Race and Inequality in Postwar Detroit. Princeton, NJ: Princeton Univ. Press

[113] Sze J. 2006. Noxious New York: The Racial Politics of Urban Health and Environmental Justice. Cambridge: MIT Press

[114] Sze J. 2020. Environmental Justice in a Moment of Danger. Oakland: Univ. Calif. Press

[115] Taylor D. 2014. Toxic Communities: Environmental Racism, Industrial Pollution, and Residential Mobility. New York: NYU Press

[116] Tessum CW, Paolella DA, Chambliss SE, Apte JS, Hill JD, Marshall JD. 2021. PM_2.5_ polluters disproportionately and systemically affect people of color in the United States. Sci. Adv 7(18):eabf4491 33910895 10.1126/sciadv.abf4491 PMC11426197

[117] Tran KV, Casey JA, Cushing LJ, Morello-Frosch R. 2021. Residential proximity to hydraulically fractured oil and gas wells and adverse birth outcomes in urban and rural communities in California (2006-2015). Environ. Epidemiol 5:e172 34909552 10.1097/EE9.0000000000000172 PMC8663888

[118] Tustin AW, Hirsch AG, Rasmussen SG, Casey JA, Bandeen-Roche K, Schwartz BS. 2017. Associations between unconventional natural gas development and nasal and sinus, migraine headache, and fatigue symptoms in Pennsylvania. Environ. Health Perspect 125:189–97 27561132 10.1289/EHP281 PMC5289909

[119] United Church of Christ Comm. Racial Justice. 1987. Toxic Wastes and Race in the United States: A National Report on the Racial and Socio-Economic Characteristics of Communities with Hazardous Waste Sites. New York: United Church of Christ

[120] U.S. Comm. Civil Rights. 2016. Environmental justice: examining the Environmental Protection Agency’s compliance and enforcement of Title VI and Executive Order 12,898. Rep., U.S. Comm. Civil Rights, Washington, DC. https://www.usccr.gov/files/pubs/2016/Statutory_Enforcement_Report2016.pdf

[121] U.S. Environ. Prot. Agency. 1994. Executive Order 12898: federal actions to address environmental justice in minority populations and low-income populations. Laws & Regulations. https://www.epa.gov/laws-regulations/summary-executive-order-12898-federal-actions-address-environmental-justice

[122] U.S. Gov. Print. Off. 1983. Siting of hazardous waste landfills and their correlation with racial and economic status of surrounding communities. GAO/RCED-83-168, U.S. Gov. Print. Off., Washington, DC. https://www.gao.gov/assets/rced-83-168.pdf

[123] Vargo J, Stone B, Habeeb D, Liu P, Russell A. 2016. The social and spatial distribution of temperature-related health impacts from urban heat island reduction policies. Environ. Sci. Policy 66:366–74

[124] Vickery J, Hunter LM. 2016. Native Americans: where in environmental justice research? Soc. Nat. Resour 29:36–52 27103758 10.1080/08941920.2015.1045644 PMC4835033

[125] Wallerstein N, Muhammad M, Sanchez-Youngman S, Rodriguez Espinosa P. 2019. Power dynamics in community-based participatory research: a multiple–case study analysis of partnering contexts, histories, and practices. Health Educ. Behav 46:19S–32S 31549557 10.1177/1090198119852998

[126] Winling LC, Michney TM. 2021. The roots of redlining: academic, governmental, and professional networks in the making of the New Deal lending regime. J. Am. Hist 108:42–69

[127] Woo B, Kravitz-Wirtz N, Sass V, Crowder K, Teixeira S, Takeuchi DT. 2019. Residential segregation and racial/ethnic disparities in ambient air pollution. Race Soc. Probl 11:60–67 31440306 10.1007/s12552-018-9254-0 PMC6706065

[128] Woo H, Brigham EP, Albright K, Ejike C, Galiatsatos P, 2021. Racial segregation and respiratory outcomes among urban Black residents with and at risk of chronic obstructive pulmonary disease. Am. J. Respir. Crit. Care Med 204:536–45 33971109 10.1164/rccm.202009-3721OC PMC8491265

